# Hypnotic Suggestion in Dental Practice*Extracted from a paper published by the British *Dental Record*, for January, 1920.

**Published:** 1920-02

**Authors:** Betts Toplan


### HYPNOTIC SUGGESTION IN DENTAL PRACTICE *

BY DR. BETTS TOPLAN

The popular idea of hypnotism is that a person is put into a deep sleep and that he knows nothing that is going on. That does not follow at all. I supose I have seen about 14,000 cases; that is, I have treated about 14,000. I should think that quite 80 per cent. of those people have heard all I have said to them, heard every word. They simply sit in my easy chair; they pass into a comfortable rest. My suggestions then tell. Supposing it were a case of asthma with very bad breathing. If I tell him, "Your breathing is getting easier. You take a deep breath from the basis of your lung," that man is breathing perfectly quietly in a quarter of an hour. And this is not only when he is in my chair, but it does not come back. He comes to me a few times more and the man is better. Supposing a person is going to have a tooth out. He is sitting in the chair perfectly comfortable. I rub his jaw gently and say: "That gum is getting quite numb, as if you have had cocaine in it." I go on for about five minutes. "It is getting more and more numb," I say. "It

*Extracted from a paper published by the British *Dental Record,* for January, 1920.

feels quite dead.'' I go on saying: ''It is quite numb—numb; you can not feel when it is touched.'' I say: ''Now when you go to your dentist tomorrow, when you sit in his chair, that numbness will come back and it will not hurt you a bit. What is more, you will not mind going a bit. You will not feel nervous.'' This is actually what happens, gentlemen; this has happened again and again. Two ladies told me at different times that they had to visit their dentists. I suggested to them that when the time came they would not feel it a bit. I rubbed the gums and said: ''When you go to your dentist you will not feel a bit nervous, and when you get there into the chair, you will not mind a bit, and when the tooth comes out it will not hurt you.'' In both instances they came back after the operations and told me it did not hurt them a bit. I remember in my own cook's case last week. She had toothache. I took her to my studio and treated her by suggestion and I stopped the toothache. I told her that when she went to the dentist she would not be at all afraid and the tooth would come out without hurting her a bit. She might feel it come out, but it would not hurt her. She went to the dentist and the dentist told me that she did not cry out. She told me that if she felt it at all it was very slight. If you rob your patients of the general state of fear and dread when they go to their dentist, you have done something. You know what a hysterical or a nervous person is in a chair. If you could make her calm, and if you could do more than that, and make her tooth come out without any exclamation or pain, what a great thing it would be! I took my daughter to a gentleman who I was hoping would be here. She had to have a tooth stopped. It was very bad. She had to have the pulp dealt with and the nerve removed. The dentist said to me, ''It is very tender. Shall I put her under cocaine?'' She sat in the chair and I said: ''Go to sleep and do not wake up till I tell you.'' For twenty or twenty-five minutes she did not take the slightest notice. He killed the nerve. When he was done he was astonished. I said to her, "When you wake up you will feel quite comfortable. When I count ten, you wake up." She said, "Is it over, daddy?" And I said, "Yes." I remember a lady who had had gas twice, I think, in her life, and she feels all the pain, or says she does, when she has gas. She was dreading the gas so frightfully that her husband got me to go and treat her. I gave her two sittings. I said to her: "When you go to the dentist's you will go quite calmly; you will not mind it a bit; and when you take the gas, you will like it; and when you wake up you will be quite well." It sounds like a fairy tale, but it is absolutely true and I can produce the dentist. She had two or three sittings. She took the gas and she liked it. I sat in the corner. The teeth were removed, four or five, and then she woke up. She had not a tenderness in her gums even. She told me it was delightful; she never saw gas so delightfully given; she thought it was the doctor who gave it, but it was not. I assure you she was the sort of person you would dread giving gas to. You could not have pulled those teeth out, even with cocaine, without a lot of trouble. Now, gentlemen, why should you not do that? I know it is a fact that everybody is not fitted for this psycho-therapeutic work; it does require a certain amount of personality or something of that kind. But there are a large number of you who can do it. The way to do it is the way I say. You want an absolutely quiet room, not even a ticking clock. Get them to go loose, to relax their muscles. Ask them to think of sleep. The simplest way and the least theatrical way would be to close the patient's eyes and tell them to think of sleep. Then open them again and say, "You will get more sleepy." Then close them again and say, "Next time you will be still more comfortable and sleepy. Think of rest and never mind what I say." If it is a case of anaesthesia without any gas, all you have got to do is to suggest to them that they will feel nothing, that they will be quite calm and happy; rub the gum gently; tell them it is getting numb; give them a quarter of an hour of that; say two or three sittings like that before the operation, and then when the time comes repeat the process. You will find that in nine out of ten cases they will not feel it, or if they do, it will be so slight they do not bother about it. It is not a thing you can learn directly; it is not a thing some people can learn at all. If you can produce slight suggestibility, you will save yourself a lot of gas, ether or chloroform, and that sort of thing. I can only leave you to get books on the subject yourselves, and to use it in your own way. You will each acquire your own method. You will find it will make a great deal of difference in your work. The late Mr. Arthur Turner, a dental surgeon of Leeds, reports cases which came under his care in which Dr. Milne Bramwell had induced hypnosis for the occasion with great and complete success. I can only point the way for you. It took me a number of years. The only way to do is to get some good work on the subject, study it carefully, study the theory, the psychology, the practical methods of the thing, and apply them yourself for your own work, and I think you will find you will be very glad you did.

